# Supplementary material for: A comparison study of temporal trends of SARS-CoV2 RNAemia and biomarkers to predict success and failure of high flow oxygen therapy among patients with moderate to severe COVID-19
Source: PLoS One. 2024 Jul 10;19(7):e0305077. doi: 10.1371/journal.pone.0305077 (PMC11236165; doi:10.1371/journal.pone.0305077)
Supplement: S1 Table — (DOCX) [file pone.0305077.s001.docx]

**S1 table: Baseline characteristics of the patients among three groups**

|  | **Success (n=105)** | **Early failure (n=6)** | **Late failure (n=11)** | ***p*-value** |
| --- | --- | --- | --- | --- |
| **Age (in years)** | 52.0 [46.0, 60.0] | 48 [40.5, 61.5] | 57 [50, 74.5] | 0.022 |
| **Sex, Female/Male, n(%)** | 25/80 (23.8/76.2) | 1/5 (16.7/83.3) | 5/6 (45.5/54.5) | 0.240 |
| **BMI, kg/m^2^** | 26.8 [24.1, 31.6] | 28.1 [27.7, 32.9] | 28.0 [23.2, 30.0] | 0.711 |
| **Current smoker, n(%)** | 18 (17.1) | 3 (50.0) | 2 (18.2) | 0.153 |
| **Underlying diseases, n(%)** |  |  |  |  |
| **Hypertension** | 33 (31.4) | 2 (33.3) | 8 (72.7) | 0.025 |
| **Diabetes** | 46 (43.8) | 2 (33.3) | 8 (72.7) | 0.161 |
| **COPD** | 4 (3.8) | 0 (0) | 0 (0) | 1.000 |
| **Asthma** | 2 (1.9) | 0 (0) | 0 (0) | 1.000 |
| **CHF** | 2 (1.9) | 0 (0) | 0 (0) | 1.000 |
| **Stroke** | 2 (1.9) | 0 (0) | 3 (27.3) | 0.010 |
| **Liver cirrhosis** | 5 (4.8) | 0 (0) | 0 (0) | 1.000 |
| **CKD** | 4 (3.8) | 0 (0) | 0 (0) | 1.000 |
| **Immunocompromised** | 1 (1.0) | 0 (0) | 1 (9.1) | 0.260 |
| **Malignancy** | 0 (0.0) | 0 (0) | 1 (9.1) | 0.139 |
| **Number of vaccinations** |  |  |  |  |
| **0** | 83 (79.0) | 5 (83.3) | 8 (72.7) | 0.658 |
| **1** | 18 (17.1) | 1 (16.7) | 2 (18.2) |  |
| **2** | 2 (1.9) | 0 (0) | 0 (0) |  |
| **not recorded** | 2 (1.9) | 0 (0) | 1 (9.1) |  |
| **Days from onset to admission (in days)** | 8.0 [7.0, 10.0] | 6.5 [5.25, 8.5] | 7.0 [6.0, 8.0] | 0.309 |
| **Vital signs** |  |  |  |  |
| **SBP, mmHg** | 130.0 [118.0, 139.0] | 145.0 [134.0, 150,0] | 132.0 [126.0, 157,0] | 0.114 |
| **HR, rate/min** | 92.0 [81.0, 101.0] | 90.0 [80.5, 94.2] | 90.0 [84.0, 102,0] | 0.841 |
| **BT, °C** | 37.6 [36.8, 38.5] | 38.1 [37.8, 38.5] | 36.9 [36.4, 37.2] | 0.037 |
| **Respiratory condition on admission** |  |  |  |  |
| **SpO_2_^,^ %** | 94.0 [92.0, 95.0] | 92.0 [88.5, 95.5] | 92.0 [88.5, 92.5] | 0.034 |
| **Respiratory rate, rate/min** | 23.0 [20.0, 28.0] | 25 [18.5, 30.0] | 23 [19.5, 25.5] | 0.926 |
| **Oxygen administered, n(%)** | 103 (98.1) | 6 (100.0) | 11 (100.0) | 1.000 |
| **FiO_2_** | 41.0 [33.0, 50.0] | 46.5 [37.8, 58.2] | 50.0 [43.0, 56.5] | 0.102 |
| **SpO_2_/FiO_2_ ratio** | 218.0 [186.0, 285.0] | 198.0 [157.0, 254,0] | 184.0 [167.0, 216.0] | 0.052 |
| **ROX index** | 9.9 [7.8, 13.2] | 8.9 [7.82, 9.52] | 8.2 [6.3, 11.4] | 0.195 |
| **Pneumonia on initial chest CT, n(%)** | 105 (100.0) | 6 (100.0) | 11 (100.0) | 1.000 |
| **Therapeutic agents until admission day 3** |  |  |  |  |
| **Remdesivir, n(%)** | 99 (94.3) | 6 (100.0) | 11 (100.0) | 1.000 |
| **Dexamethasone, n(%)** | 105 (100.0) | 6 (100.0) | 11 (100.0) | 1.000 |
| **Baricitinib, n(%)** | 76 (72.4) | 2 (33.3) | 9 (81.8) | 0.096 |
| **Casirivimab/Imdevimab, n(%)** | 6 (5.7) | 0 (0) | 0 (0) | 1.000 |

Notes: Continuous variables are presented as medians with interquartile ranges in square brackets and were analyzed using the Kruskal–Wallis test. Categorical variables are presented as numbers and percentages in parentheses and were analyzed using Fisher’s exact test.

Abbreviations: HFNC, high flow nasal cannula; BMI, body mass index; COPD, chronic obstructive pulmonary disease; CHF, chronic heart failure; CKD, chronic kidney disease; SBP, systolic blood pressure; HR, heart rate; BT, body temperature; SpO_2_, peripheral capillary

oxygen saturation; FiO_2_, fraction of inspired oxygen; ROX, respiratory rate-oxygen; CT, computed tomography.
