# Supplementary material for: A comparison study of temporal trends of SARS-CoV2 RNAemia and biomarkers to predict success and failure of high flow oxygen therapy among patients with moderate to severe COVID-19
Source: PLoS One. 2024 Jul 10;19(7):e0305077. doi: 10.1371/journal.pone.0305077 (PMC11236165; doi:10.1371/journal.pone.0305077)
Supplement: S2 Table — (DOCX) [file pone.0305077.s002.docx]

**S2 Table.** **Trends in each laboratory parameter and SARS-CoV2 RNAemia**

| **Laboratory parameters** | **Day 1* (n=122)** | | | **Day 4 (n=116)** | | | **Day 7 (n=111)** | | |
| --- | --- | --- | --- | --- | --- | --- | --- | --- | --- |
|  | **Success**  **(n=105)** | **Failure**  **(n=17)** | ***p*-value** | **Success**  **(n=105)** | **Failure**  **(n=11)** | ***p*-value** | **Success**  **(n=104)** | **Failure**  **(n=7)** | ***p*-value** |
| **NL ratio** | 7.61  [5.13–10.79] | 11.24  [6.41–14.83] | 0.093 | 8.82  [6.13–11.72] | 9.91  [8.22–16.40] | 0.13 | 6.70  [4.39–12.43] | 25.14  [17.00–35.65] | <0.001 |
| **CRP, mg/dL** | 11.68  [7.69–6.86] | 10.29  [6.74–13.96] | 0.408 | 1.95  [1.16–3.42] | 1.14  [0.80–2.11] | 0.049 | 1.04  [0.47–1.89] | 1.35  [0.83–1.75] | 0.561 |
| **IL-6, pg/mL** | 69.45  [46.33–108.50] | 123.00  [64.05–170.00] | 0.102 | 8.65  [5.67–15.17] | 27.30  [12.77–33.15] | 0.003 | 10.65  [6.25–16.42] | 30.30  [19.50–42.40] | 0.003 |
| **LDH, U/L** | 549.0  [456.0–690.0] | 627.0  [531.0–782.0] | 0.082 | 465.0  [381.0–535.0] | 626.0  [560.0–723.5] | 0.001 | 410.0  [332.5–475.0] | 636.0  [573.5–793.5] | <0.001 |
| **Ferritin, ng/mL** | 982.3  [371.7–1748.8] | 1254.6  [689.3–2357.0] | 0.234 | 1130.8  [615.9–2005.6] | 1993.4  [1057.1–2579.6] | 0.119 | 840.1  [542.3–1527.4] | 1687.9  [893.7–2073.0] | 0.105 |
| **D-dimer, μg/ml** | 0.80  [0.50–1.33] | 1.40  [0.80–1.50] | 0.049 | 1.10  [0.70–2.10] | 1.40  [0.85–2.95] | 0.431 | 1.60  [0.80, 2.80] | 7.00  [1.95–15.15] | 0.041 |
| **SARS-CoV2 RNAemia†** |  |  |  |  |  |  |  |  |  |
| **not detected/detected, n(%)** | 57/35  (62.0/38.0) | 2/13  (13.3/86.7) | 0.001 | 67/33  (67.0/33.0) | 0/11  (0.0/100.0) | <0.001 | 82/14  (85.4/14.6) | 2/5  (28.6/71.4) | 0.001 |
| **log RNA, copies/μL** | 0.00  [0.00–3.65] | 9.70  [4.70–15.95] | <0.001 | 0.00  [0.00–1.48] | 2.38  [2.04–3.28] | <0.001 | 0.00  [0.00, 0.00] | 1.86  [0.79, 2.17] | <0.001 |

Abbreviations: NL, neutrophil-lymphocyte; CRP, C-reactive protein; IL-6, interleukin-6; LDH, lactate dehydrogenase; SARS-CoV2, severe acute respiratory syndrome coronavirus 2.

Data were presented as median [interquartile range] unless otherwise indicated.

Missing values: NL ratio, n=1 on day 1, n=1 on day 7; CRP, n=8 on day 4, n=2 on day 7; IL-6, n=15 on day1, n=5 on day 4, n=8 on day 7; LDH, n=1 on day 7; ferritin, n=13 on day 1, n=1 on day 7; D-dimer, n=1 on day 1, n= 1 on day 1; SARS-CoV2 RNA, n=15 on day 1, n=5 on day 4, n=8 on day 7.

*Day 1 refers to the day of admission.
