# Supplementary figures and images for: A comparison study of temporal trends of SARS-CoV2 RNAemia and biomarkers to predict success and failure of high flow oxygen therapy among patients with moderate to severe COVID-19
Source: PLoS One. 2024 Jul 10;19(7):e0305077. doi: 10.1371/journal.pone.0305077 (PMC11236165; doi:10.1371/journal.pone.0305077)

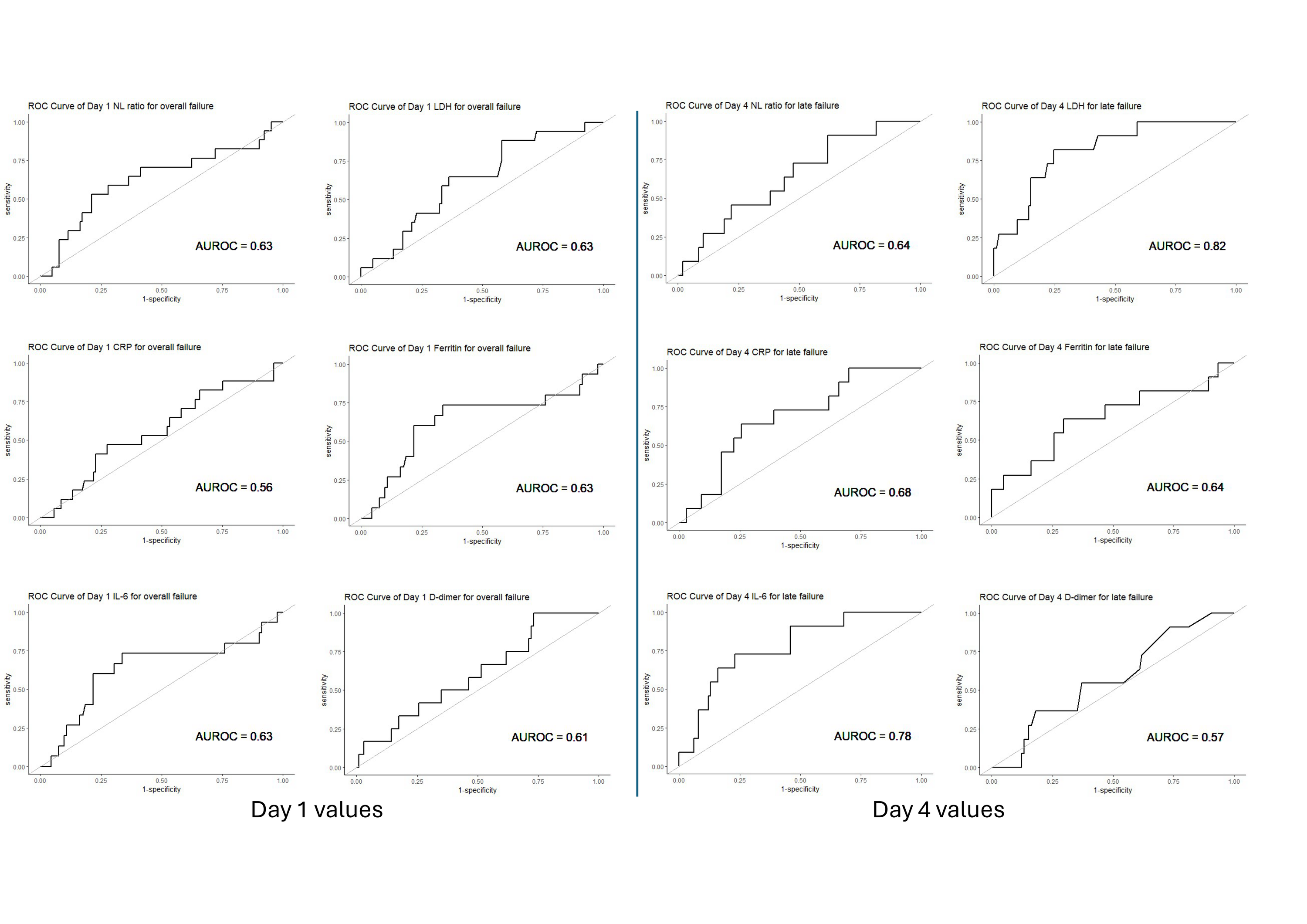

Supplement: S1 Fig — The left-hand side of the graph depicts the AUROC for overall HFNC failure for each laboratory marker on Day 1, whereas the right-hand side shows the AUROC for late HFNC failure for each laboratory marker on Day 4. (TIF) [file pone.0305077.s003.tif]

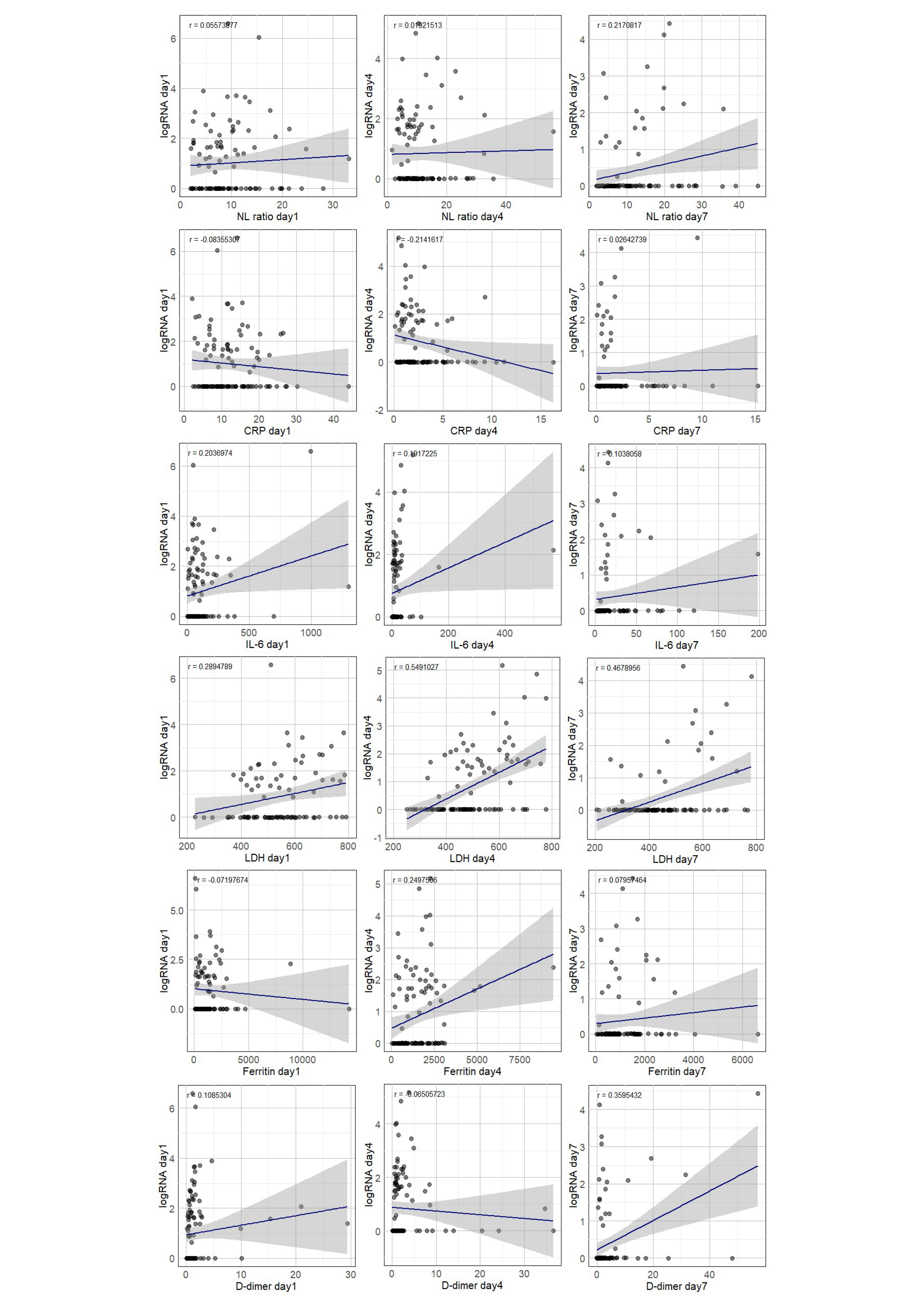

Supplement: S2 Fig — The scatter plot and regression line with a 95% confidence interval (shown in gray) shows a correlation between log-transformed SARS-CoV2 RNA level (vertical axis) and laboratory parameters on Days 1, 4, and 7 (horizontal axis), with the Pearson correlation coefficient (r) indicating the strength and direction of the correlation. (TIF) [file pone.0305077.s004.tif]
